# Naturalist's Monthly Report

**Published:** 1811-02

**Authors:** 


### NATURALIST'S MONTHLY REPORT.

### DECEMBER.

### *Dead Winter Month.*

————————Now joyless rains obscure

Drive through the mingling skies with vapour foul;

Dark on the mountain's brow, and shake the woods.

During nearly the whole of the present month the weather has been as variable as I almost ever recollect it. On the first and second the wind was Northerly, accompanied with sharp frost. From the 3d to the 6th it was westerly; North West from the 7th to the 9th; South-West on the 10th; North North East on the 11th; Westerly from the 12th to the 15th; North on the 16th and 17th; South West and North West on the 18th; Easterly on the 19th; South West and West

West on the 20th ; Westerly from the 21st to the 24th ; North West from the 25th to the 27th ; Northerly on the 28th and 29th ; and North East on the 30th and 31st.

We had strong gales from South West or North West on the 6th, 18th, 21st, 23d, 25th, and 27th, and hard gales on the 12th and 14th. The latter was a tremendous day.

There has been rain on sixteen days of this month, but on the 3d, 6th, 10th, 12th, 18th, 20th, and 22d, much more than on any of the others. A hard frost commenced in the night of the 28th, and continued till the end of the month. In the night of the 31st there was a considerable fall of snow, the first we have had this year.

December 1.—The season has hitherto been so mild that several of the field flowers are yet in bloom. Among them I observe the hedge Lychnis, (*Lychnis dioica*), common fumitory, (*fumaria officinalis*) and Gorse.

December 6.—A great quantity of herrings were caught in the evening of this day ; and to the westward of this neighbourhood, herrings have continued to be caught during the greater part of the month.

December 7.—The weather was so warm that a large blue fly came in at the window of my sitting room, and buzzed about upon the glass in the same manner as these flies do in summer.

No pilchards have hitherto, this year, migrated so far eastward as to our shores.

December 13.—Ewes have yeaned some days ago, and lambs are now, in several places, to be seen in the fields.

December 16.—So warm does the weather still continue, that a snake was this day seen out of its hole; and in the evening I observed black beetles of various species (*scarabeus stercorarius, &c.*) flying about in every direction.

December 17.—Spiders appear upon their webs, and seem to be unaffected by the lateness of the season. The black long legged insects which run upon the surface of the water, and are usually denominated by the common people, water spiders (*Cimex lacustris* and *stagnorum* of Linnæus) continue to be seen.

Bats are still to be seen flitting about in the evenings.

December 21.—The following plants are in flower, sweet-scented violet, wall-flower, mezereon, and hepatica.

Snipes have in a great measure left the marshes, and are found on the dry lands.

December 25 and 27.—In the evening of each of these days there was much lightning.

December 31.—No wild fowl, except a very few ducks and geese, have yet visited us. The variable weather has no doubt been the cause of this. A tolerably severe frost with the wind from the eastward, are the usual prognostics of the arrival of these birds.

*Hampshire.*

METEORO-

